# Freezing of gait in Parkinson’s disease: pathophysiology, risk factors and treatments

**DOI:** 10.1186/s40035-020-00191-5

**Published:** 2020-04-15

**Authors:** Chao Gao, Jun Liu, Yuyan Tan, Shengdi Chen

**Affiliations:** 1 Department of Neurology, Ruijin Hospital Affiliated to Shanghai Jiao Tong University School of Medicine, Shanghai, China; 2 Co-innovation Center of Neuroregeneration, Nantong University, Nantong, Jiangsu Province China

**Keywords:** Freezing of gait, Parkinson’s disease, Pathophysiology, Risk factor, Pharmacological treatment, Non-pharmacological treatment

## Abstract

**Background:**

Freezing of gait (FOG) is a common, disabling symptom of Parkinson’s disease (PD), but the mechanisms and treatments of FOG remain great challenges for clinicians and researchers. The main focus of this review is to summarize the possible mechanisms underlying FOG, the risk factors for screening and predicting the onset of FOG, and the clinical trials involving various therapeutic strategies. In addition, the limitations and recommendations for future research design are also discussed.

**Main body:**

In the mechanism section, we briefly introduced the physiological process of gait control and hypotheses about the mechanism of FOG. In the risk factor section, gait disorders, PIGD phenotype, lower striatal DAT uptake were found to be independent risk factors of FOG with consistent evidence. In the treatment section, we summarized the clinical trials of pharmacological and non-pharmacological treatments. Despite the limited effectiveness of current medications for FOG, especially levodopa resistant FOG, there were some drugs that showed promise such as istradefylline and rasagiline. Non-pharmacological treatments encompass invasive brain and spinal cord stimulation, noninvasive repetitive transcranial magnetic stimulation (rTMS) or transcranial direct current stimulation (tDCS) and vagus nerve stimulation (VNS), and physiotherapeutic approaches including cues and other training strategies. Several novel therapeutic strategies seem to be effective, such as rTMS over supplementary motor area (SMA), dual-site DBS, spinal cord stimulation (SCS) and VNS. Of physiotherapy, wearable cueing devices seem to be generally effective and promising.

**Conclusion:**

FOG model hypotheses are helpful for better understanding and characterizing FOG and they provide clues for further research exploration. Several risk factors of FOG have been identified, but need combinatorial optimization for predicting FOG more precisely. Although firm conclusions cannot be drawn on therapeutic efficacy, the literature suggested that some therapeutic strategies showed promise.

## Background

Freezing of gait (FOG) was defined as “brief, episodic absence or marked reduction of forward progression of the feet despite the intention to walk” [1, 2]. Patients suddenly feel as if their feet are glued to the ground when they try to move forward. Typically, FOG lasts a couple of seconds, but this transient episode can occasionally exceed 30 s [3]. In rare circumstances, the patient is unable to generate any effective steps for several minutes or even longer, until compensation strategies such as cueing is provided. FOG leads to significant falls in patients with Parkinson’s disease (PD) [4], which reduces patients’ independence and mobility profoundly, and significantly impairs their quality of life [5].

Our understanding towards mechanism in FOG remains incomplete, and treatment of FOG is perceived by clinicians as a very challenging task. Several hypotheses have been proposed to explain the freezing phenomenon and many approaches have been applied to treat FOG. In this review, firstly, we summarized the physiology of gait control, and briefly introduced the mechanism hypotheses of FOG. Secondly, we summarized the risk factors of FOG to facilitate FOG screening in consideration of early therapeutic interventions to delay or even prevent the onset of FOG. Thirdly, various treatment approaches exist, including pharmacological and non-pharmacological treatments. Non-pharmacological treatments including invasive brain and spinal cord stimulation, noninvasive brain and vagus nerve stimulation, and physiotherapeutic approaches. Clinical trials involving various therapeutic strategies were summarized in this review. In addition, the limitations and recommendations for future research design were also discussed.

## Main text

### Possible mechanisms underlying FOG

The main supraspinal regions exerting critical roles in locomotion are pontomedullary reticular formation (PMRF), mesencephalic locomotor region (MLR), basal ganglia, cerebellum, and cerebral cortex [6]. MLR receives inputs from basal ganglia and cortex neurons, projects to PMRF to initiate and modulate the spinal neural circuitry to control posture and gait [7]. The major nucleis of the MLR are pedunculopontine nucleus (PPN), cuneiform nucleus and subcuneiform nucleus, in which PPN is particularly important for gait [8]. The basal ganglia receives inputs from cerebral cortex and controls voluntary motor movements through GABAergic projections to other brain regions. The primary motor cortex mainly projects to the spinal cord, and lesions in the primary motor cortex could cause contralateral paralysis. The supplementary motor area (SMA) and premotor area (PM) are also indispensable for locomotion control. The SMA and PM have dense projections to both the spinal cord and the brainstem reticular formation, which are associated with movement initiation [9]. In addition, sensation information processing and integration in the cortex are important as well. Only when the temporoparietal cortex integrates visual, proprioceptive, and vestibular sensations timely and correctly can the PM and SMA generate the motor program accurately, particularly when encountering an unfamiliar environment [10].

Several hypotheses have been proposed based on the FOG phenotype and mechanistic studies. Nieuwboer et al. [11] summarized the pathological mechanisms in the following four models: a) The threshold model [12]: Compared with non-freezers, patients with FOG present significant gait disorders such as reduced stride amplitude, impaired gait coordination and increased variability of step timing. This model assumes that when these motor deficits accumulate to a point of motor breakdown, then FOG occurs. b) The interference model [13]: The relationship of motor, cognitive and limbic circuits is proposed as both competing and complimentary. As dopamine neurons are mostly depleted in PD patients, concurrent processing of cognitive and/or limbic information during motor task will overload the information processing capacity within basal ganglia, thus leading to disordered neuronal crosstalk between these circuits. The interference between neural circuits would explain the phenomena that increasing cognitive load while performing a dual task will break down the locomotion. c) The cognitive model [14]: This modal emphasized the conflict-resolution deficit, which is one aspect of executive dysfunction. Under normal condition, people will prevent premature action and delay the response selection until resolving the conflict. In contrast, patients with FOG fail to process response conflict, impose faster response decision but with greater incongruence, thus FOG is triggered. d) The decoupling model [15]: This model regarded FOG as a disconnection between pre-planned motor program and motor response. These models and hypotheses explained FOG from various perspectives. Albeit incomplete and can only partially interpret the pathophysiology and clinical phenotype of FOG, it is worth noting that these models are helpful for better understanding and characterizing FOG, and provide clues for further research exploration. More importantly, based on these models, behavior strategies can be invented and implemented for FOG treatment.

### Risk factors for developing FOG

This review included the prospective studies that followed early-stage PD patients over time and the retrospective studies that clearly recorded the clinical manifestation prior to FOG onset. Many studies identified FOG related motor and non-motor symptoms by utilizing cross-section data to compare the clinical symptoms between freezers and non-freezers. Those studies were excluded as they cannot differentiate the symptoms prior to FOG from the accompanying symptoms of FOG. A total of 11 longitudinal follow-up studies were reviewed and summarized (Table 1). For these studies, cox proportional-hazards regression analyses [16, 17, 20, 26] or logistic regression analysis [18, 19, 22–25] were performed to identify the factors predictive of FOG except one study that used generalized estimating equations [21]. Here, we elaborated the risk factors including demographic information, motor symptoms, non-motor symptoms, neuroimaging, fluid parameters, and medication use. Current limitations and recommendations for future researches will also be discussed. A list of the evidence supporting and refuting the following variables as risk factors of FOG was provided in Table 2.

**Table 1:** A detail summary of risk factors for FOG development in Parkinson’s disease

Main findings	Participants	Assessment	Follow-up duration	FOG define	Ref
CSF Aβ42 was a predictor of FOG in patients with early PD. PIGD score, caudate DAT uptake, and CSF Aβ42 together could predict FOG within 4 years after diagnosis of PD (AUC = 0.755).	PPMI database (393 early do novo PD without FOG at baseline)	CSF (Aβ42, α-synuclein, t-tau, p-tau, Aβ42/ t-tau); motor; non-motor (olfactory, sleep, cognition, depression, anxiety, autonomic function); DAT imaging (striatal region)	Median 4.0 years	MDS-UPDRS item 2.13 or item 3.11 ≥ 1	[16]
DAT uptakes in the caudate nucleus and putamen predicted the development of FOG. Male sex, higher PIGD score, and lower MoCA score were also significant predictors of FOG.	PPMI data (390 early do novo PD without FOG at baseline)	DAT imaging (striatal region); motor; cognition	Median 4.0 years	MDS-UPDRS item 2.13 or item 3.11 ≥ 1	[17]
FOGQ total score and the anxiety score were the strongest predictors, and using only these two factors could significantly predict FOG in the next 15 months with 82% accuracy.	221 PD in which 88 patients had FOG at baseline	Motor; non-motor (cognition, anxiety, depression, sleep); medication use	Mean 14 months	FOGQ item 3 ≥ 1	[18]
Depressive, gait speed and UPDRS-III (off vs. on) were the independent predictors of future FOG.	57 PD patients without FOG at baseline	Motor; non-motor (sleep, cognition, autonomic, depression, and others); medication use	Mean 5 years	NFOGQ (item1 = 1) and objective observation	[19]
Increased risk: Onset of PD with a gait disorder; higher scores of rigidity, postural instability, bradykinesia and speech; and longer disease duration; absence of tremor. Decreased risk: Deprenyl treatment	DATATOP data (800 early PD in which 57 patients had FOG at study entry)	Motor; non-motor (speech, cognition, depression); initial symptoms	Mean 14 ± 5 months	UPDRS II-item14 ≥ 1	[20]
Motor fluctuations, higher levodopa dose were independent risk factors; none of the cardinal features independently predicted FOG.	232 PD without FOG at baseline	Motor and motor complication; non-motor (psychosis (UPDRS I item2, hallucinations or delusions), cognition); medication use	12 years	UPDRS II-item14 ≥ 1	[21]
Lower education, akinetic-rigid style, not using dopamine receptor agonists, sleep disorders (insomnia); cognitive disturbances were predictors of FOG.	248 early PD without FOG at baseline	Motor; non-motor (anxiety; depression); medication use;	3 years	FOGQ item 3 ≥ 1 and objective observation	[22]
Longer disease duration, visuospatial function deterioration, onset in lower limbs, presence of festination, falls, and hallucinations were independent predictors of FOG.	225 PD without FOG at baseline	Motor (including festination and fall); non-motor (anxiety, depression, cognition); medication use;	3 years	FOGQ item 3 ≥ 1 and objective observation	[23]
Baseline processing speed, learning and daytime sleepiness were predictive of FOG.	PPMI database (50 PD + FOG, and 50 PD-FOG at the fourth year)	Motor; non-motor (cognition, anxiety, depression, sleep)	4 years	MDS-UPDRS item 2.13 ≥ 1	[24]
A more severe depletion of presynaptic dopamine (low 123I-FP-CIT binding in the putamen and striatum) in early PD predicted FOG	41 early PD without FOG at baseline	Motor (including falls) and motor complication; DAT imaging (striatal region); medication use	9.51 ± 3.18 years	UPDRS II-item14 ≥ 1	[25]
PD group with moderate to severe WMH showed a higher risk of developing FOG (HR, 3.29; 95% CI, 1.79–6.05; *P* < 0.001) than the PD patients with minimal WMH.	268 patients with de novo PD without FOG	MRI WMH; motor (UPDRS-III, phenotype); non-motor (olfactory, cognition, depression) DAT imaging; medication; vascular risk factors	> 3 years	Inquiry and observation	[26]

A total of 11 longitudinal follow-up studies were reviewed and summarized. Main findings, recruited participants and their FOG define criteria, assessment parameters, follow-up duration were all included

*CSF* Cerebrospinal fluid, *FOG* Freezing of gait, *PD* Parkinson’s disease, *PIGD* Postural instability and gait difficulty, *DAT* Dopamine transporter, *AUC* Area under the ROC curve, *PPMI* Parkinson’s Progression Markers Initiative, *MDS-UPDRS* Movement Disorder Society-Sponsored Revision of the Unified Parkinson’s Disease Rating Scale, *MoCA* Montreal Cognitive Assessment, *FOGQ* Freezing of Gait-Questionnaire, *NFOGQ* New Freezing of Gait Questionnaire, *DATATOP* Deprenyl and tocopherol antioxidative therapy of parkinsonism, *WMH* White matter hyperintensitie

**Table 2:** A list of the evidence supporting and refuting the following variable as a risk factor of freezing of gait

	Supporting evidence^a^	Refuting evidence^a^
**Demographic risk factors**		
Male sex	[16, 17, 25]	[18–24, 26]
Low education level	[22]	[19, 23, 24]
Onset age		[16, 17, 20–23, 25, 26]
Age		[18, 19, 21–25]
Baseline longer disease duration	[20, 23]	[16–19, 21]^b^
**Motor symptoms**		
Gait disorders	[16–21, 23]	[22]
Motor phenotype	[16, 17, 21, 22]	[18, 19, 25]
Motor fluctuation	[21]	[23]
Balance, festination and falls	Festination and falls [23], balance [20]	Balance [19]
**Non-motor symptoms**		
Cognitive disturbance	[16, 17, 22–24]	[18–21]
Depression	[19, 20, 22]	[16, 23, 24]
Anxiety	[18]	[16, 23, 24]
Sleep	Insomnia [22], daytime sleepiness [24]	RBD [16, 18, 24], daytime sleepiness [16, 23]
Others	Speech problems [20], hallucination [23]	
**Neuroimaging and fluid parameters**		
Lower striatal DAT update	[16, 17, 25]	
White matter hyperintensities	[26]	
CSF Aβ42	[16]	
**Medication use**		
High LEDD	[21]	[18, 19, 23, 26]
Dopamine agonist		[22]

Demographic information, motor symptoms, non-motor symptoms, neuroimaging, fluid parameters, and medication use were all included

*DAT* Dopamine transporter, *CSF* Cerebrospinal fluid, *LEDD* Levodopa equivalent daily doses

^a^Only the prospective studies that followed early-stage PD patients over time and the retrospective studies that clearly recorded the clinical manifestation prior to FOG onset were cited here

^b^Baseline disease duration instead of the whole disease duration was analyzed in those cited studies. Relatively short disease duration might be the reason for failure to identify disease duration as a risk factor

### Demographic risk factors

#### Male sex

Two studies using the Parkinson’s Progression Markers Initiative (PPMI) data included 390 (393) patients with newly diagnosed PD at baseline [16, 17]. During a median follow-up of 4.0 years, male sex was found to be an independent risk factor of FOG (Hazard ratio (HR) = 1.512, *P* = 0.046) [16]. This finding is consistent with another study that included 41 patients with a mean follow-up disease duration of 9.51 ± 3.18 years [25]. Of these 41 PD patients, 15 patients developed FOG at the end of follow-up, in which 14 patients were males. Besides these follow-up studies, a previous cross-section study that analyzed 6620 PD patients also found that males were more likely to have FOG than females (Odds ratio (OR) = 1.19, *P* = 0.011) [27]. A number of studies have reported the existence of gender difference on specific motor or non-motor symptoms in PD. Estrogen may play a protective role in PD by influencing dopamine synthesis and release or modulating dopamine receptor expression and function [28]. However, this finding was not observed in some other studies [18–24, 26].

#### Low education level

Zhang et al’s study followed 248 early PD patients without FOG at baseline for 3 years and found that patients with a lower education level (≤9 years of education) were more prone to have FOG (OR = 0.012, *P* < 0.001) [22]. This study provided an explanation that patient with a higher education had a better understanding of PD and better compliance, leading to better treatments. However, this finding was not found in other three follow-up studies [19, 23, 24]. Although cognitive impairment is involved in the pathogenesis of FOG, whether level of education can be used as an indicator of cognitive reserve is still under debate [29].

#### Onset age and age

Eight studies recorded the age of onset and showed that there was no relationship between the age of onset and FOG development [16, 17, 20–23, 25, 26]. Seven studies recorded age at baseline [18, 19, 21–25], in which only one study showed that older age was a risk factor of FOG, but the statistical significance was weak (*P* = 0.054) [18]. Two previous cross-sectional surveys including 6620 and 683 PD patients respectively also found that age was not the predictor of FOG in PD [27, 30].

#### Longer disease duration

It is well recognized that the duration of the disease is a crucial risk factor of FOG. FOG tends to occur in the later stages of PD. Early onset FOG should be suspected of other parkinsonism, such as progressive supranuclear palsy [31]. In a retrospective study of 800 patients with early PD, only 7.1% of patients experienced FOG [20]. However, with prolonged disease progression and disease duration, FOG can affect 53% of patients in the advanced stages of the disease [32]. A previous prospective study [20] and several cross-sectional studies [27, 33, 34] also found that patients with longer disease duration were more likely to develop FOG episodes. However, other studies failed to identify disease duration as a risk factor of FOG [16–19, 21]. The reason might be that the baseline disease duration instead of the whole disease duration till the end of follow-up were analyzed between non-freezers and transitional freezers, and most patients included were early PD patients who had relatively short disease duration at baseline.

### Motor symptoms

#### Gait disorders

The Freezing of Gait-Questionnaire (FOGQ) total [18](OR = 2.34, *P* = 0.01), gait speed (on state) assessed by wearable sensors (OR = 0.01, *P* = 0.032) [19], lower limbs as the onset site (OR = 2.632, *P* = 0.013) [20, 23] were found to be risk factors of FOG, respectively. In addition, several studies found that Movement Disorder Society-Sponsored Revision of the Unified Parkinson’s Disease Rating Scale (MDS-UPDRS) motor score especially postural instability and gait difficulty (PIGD) score could predict FOG development [16, 17, 21]. These findings indicated that the gait impairment had already existed even patients didn’t present freezing episodes at the early stage of disease. This can be explained by the pathophysiology of gait control, which has been reviewed previously [8, 10, 35]. Lesions in any part of gait control system may cause abnormal gait performance. When gait impairment involved the key regions particularly important for gait, such as PPN, or when compensation was not achieved with disease progression, patients would suffer from FOG.

#### Motor phenotype

Postural instability and gait difficulty (PIGD) was identified as a risk factor of FOG in PD patients [16, 17, 21]. PD patients with PIGD not only showed more severe striatal dopaminergic terminal loss, but also presented with extrastriatal non-dopaminergic denervation compared to patients without FOG [36]. Additionally, PIGD symptoms were apparently associated with grey matter atrophy in motor-related regions and decreased functional connectivity [37]. Cognitive impairment was also significantly correlated with PIGD phenotype [38]. These mechanisms might all contribute to FOG development. However, another study did not show the difference in PIGD phenotype between non-freezers and transitional freezers at baseline [19]. If motor phenotype was classified as tremor dominant and akinetic rigid phenotype, results were controversial [22, 25]. In Zhang et al’s study, patients with akinetic-rigid phenotype were more likely to suffer from FOG 3 years later (OR = 4.881, *P* = 0.024) [22]. However, Djaldetti R et al’s study found no difference of motor phenotype between non-freezers and transitional freezers at baseline [25]. Ehgoetz et al. classified motor phenotype as tremor-dominant (TD) or non-TD phenotype, and didn’t reveal that the motor phenotype was a risk factor of FOG [18].

#### Motor fluctuation

Motor fluctuation was found to be a risk factor of FOG in Forsaa’s study. This study included 232 PD patients without FOG at baseline and found that non-freezers who presented with motor fluctuations at study entry were at more than 3-fold increased risk to develop incident FOG during the 12-year follow-up period as compared with patients without motor fluctuations at baseline [21]. Another study included 225 PD patients and did not reveal didn’t reveal motor fluctuation as a risk factor of FOG, but found that frequency of motor fluctuation at baseline was much higher in transitional freezers than non-freezers (20% vs. 10%, *P* = 0.056) [23]. Motor fluctuations are supposed to be more closely related to the severity of dopamine depletion in the basal ganglia [39], which can partly explain the phenomenon that motor fluctuation may precede the onset of FOG.

#### Balance, festination and falls

Postural control and balance [40, 41] were affected more significantly in PD patients with FOG compared with non-FOG group. FOG often occurs when turning also suggests that the postural control impairment probably contributes to freezing. However, whether balance impairment is an accompanying symptom of FOG or a risk factor of FOG remains unclear. In Giladi N et al’s study, balance was assessed by two UPDRS subitems, and they found balance impairment predicted FOG development (HR = 1.94, *P* = 0.0005) [20]. However, Herman T et al. using Berg balance scale and Activities-specific balance confidence scale to assess balance and fear of falling failed to confirm balance impairment as a risk factor in logistic regression model, although transitional freezers had significantly worse Berg balance scale score than non-freezers at baseline [19]. For festination and falls, only one study reported that presence of festination or falls at baseline was risk factors of FOG [23]. The potential mechanism underlying the relationship between gait festination and freezing in PD has been reviewed and discussed in previous reviews [42, 43]. Briefly, festination is hypothesized to be caused by the progressive delays in the timing of phasic motor cues from the internal globus pallidus to the SMA and PM. Thus the step becomes shorter and shorter. When the defect reaches the limit presenting with extremely slow or absence of the phasic cues, the motor cortical regions are not provided with phasic cues necessary to enable them to generate force for the next step in sequence, leading to the subsequent motor block-FOG.

### Non-motor symptoms

#### Cognitive disturbance

Cognitive impairment as an independent risk factor of FOG has been found by several studies [16, 17, 22]. The cognitive function domains with predictive value for FOG include baseline processing speed, learning ability [24] and visuospatial/executive abilities [23]. The involvement of cognitive impairment in FOG pathophysiology has been explained by previously proposed cognitive model [11, 14]. This model emphasized conflict-resolution deficit in executive dysfunction. Under normal condition, people will prevent premature action and delay the response selection until resolving the conflict. In contrast, when patients fail to process response conflict, impose faster response decision but with greater incongruence, FOG is triggered. Additionally, the beneficial effect of cognitive training for FOG also reflected that the pathophysiological mechanism of FOG linked to cognitive dysfunction [44]. Future studies would better enroll the PD patients without FOG at baseline, and test whether cognitive training can prevent or delay FOG development. It is worth noting that although much evidence supported cognitive impairment was a risk factor of FOG, there were still several studies didn’t confirm this finding [18–21].

#### Depression and anxiety

One study including 57 PD patients without FOG at baseline showed that depression was a strong risk factor for FOG development (OR = 10.93, *P* = 0.003). Eighty percent of the subjects who had marked depressive symptoms at baseline (Geriatric Depression Scale≥5) developed FOG at mean 5 years’ follow-up. In contrast, only 27% of those with few depressive symptoms at baseline became freezers (*p* < 0.001) [19]. A longitudinal study recruited 221 PD patients with over 1 year follow-up found that the anxiety score and FOGQ total score were the strongest predictors, and these two factors could significantly predict FOG in the next 15 months with 82% accuracy [18]. Ehgoetz et al. used the virtual reality-based functional magnetic resonance imaging approach also confirmed the association of limbic network with FOG. They found that during freezing, the coupling between limbic network (cortical and subcortical) and ventral striatum, and the coupling between limbic and cognitive control network were increased compared to normal foot tapping. In contrast, anti-coupling between the cortical and subcortical limbic network and within the subcortical network was found during freezing [45]. However, other studies did not reveal depression or anxiety as a risk factor of FOG, but found the depression or anxiety score was significantly higher at baseline in transitional freezers than non-freezers [16, 23].

#### Sleep

Kim et al’s study using PPMI database found that REM Sleep Behavior Disorder (RBD) screening questionnaire (RBDSQ) score was higher in transitional freezers compared with non-freezers, and univariable analysis also confirmed that RBD may increase the risk for FOG development. However, multivariable analysis in the same study failed to establish its risk factor effect [16]. Although gait disorders have been found in polysomnography-confirmed RBD patients [46, 47] and it was hypothesized that FOG and RBD might share a common physiopathology [48], the relationship between RBD and FOG was not confirmed [16, 18, 24]. In other two studies, RBDSQ at baseline didn’t show significant difference between transitional freezers and non-freezers [18, 24], which was supported by a previous finding that there was no freezing difference between the patients with probable RBD and without RBD [49]. Other studies explored the relationship of insomnia or daytime sleepiness with FOG. One study demonstrated that insomnia assessed by Hamilton Depression Rating Scale (OR = 2.418, *P* = 0.036) was an independent risk factor for FOG development [22]. Three studies evaluated daytime sleepiness, but the results were inconsistent [16, 23, 24]. In Bank et al’ s study, daytime sleepiness assessed by Epworth Sleepiness Scale (ESS) was identified as a risk factor of future FOG [24]. However, Kim et al’ s study failed to establish its risk factor effect, although they found that ESS score was higher in transitional freezers compared with non-freezers, and ESS may increase the risk for FOG development in univariable analysis [16]. Another study didn’t find the difference of daytime sleepiness between non-freezers and transitional freezers at baseline [23].

#### Others

Speech disturbance [50] was more frequent in patients with FOG. The DATATOP (Deprenyl and tocopherol antioxidative therapy of parkinsonism) study also found that patients with speech problems at baseline were more likely to develop FOG at the end of follow-up [20]. Another study evaluated hallucination using UPDRS part I subitem and found that hallucination was a risk factor of FOG [23]. These findings need to be confirmed by future studies and which type of speech disorder and hallucination related to FOG needs to be evaluated.

### Neuroimaging and fluid parameters

#### Striatal DAT update

Several studies have found that presynaptic striatal dopaminergic depletion examined by dopamine transporter (DAT) uptake predicted the later development of FOG in de novo PD [16, 17, 25]. Caudate DAT uptake was more significantly related to FOG development compared with putamen [16]. The combined model integrating the caudate DAT uptake, PIGD score, and cerebrospinal fluid (CSF) Aβ42 together can predict FOG within 4 years after diagnosis of PD (area under the curve 0.755, 95% CI 0.700–0.810). These findings demonstrated a direct association between the initial presynaptic striatal dopamine loss and the subsequent development of FOG in patients with early PD.

#### White matter hyperintensities (WMH)

Recently one study retrospectively reviewed medical records of 268 patients with de novo PD (follow-up > 3 years), and evaluated the longitudinal effects of WMH on the development of FOG using cox regression model [26]. Results showed that after adjusting for age, sex, striatal DAT availability and levodopa equivalent dose, the PD group with moderate to severe WMH showed a higher risk of developing FOG (HR = 3.29, *P* < 0.001) than the PD patients with minimal WMH. This study was consistent with previous studies which showed that increased WMH burden was associated with severe motor deficits, especially axial motor impairments [51, 52]. Although the mechanisms linking WMH and motor disability are not fully understood, diffuse white matter damage including major cortico-cortical, motor-related cortico-fugal and several striato-frontal tracts are involved in the gait control network, which may contribute to FOG development.

#### CSF Aβ42

One recent study [16] using the PPMI data included 393 newly diagnosed PD patients without FOG at baseline aimed to explore whether CSF biomarker changed prior to FOG occurrence. Beta-amyloid 1-42 (Aβ42), α-synuclein, total tau, phosphorylated tau, and the calculated ratio of Aβ42 to total tau in CSF at baseline was evaluated. Cox proportional-hazards regression analysis was performed to identify factors predictive of FOG. They found that in multivariable Cox analysis, only Aβ42 of those CSF biomarkers was associated with the development of FOG (HR = 0.997, *P* = 0.009). This study proposed the possible mechanisms underlying Aβ and FOG. Firstly, Aβ plaque influences the neural circuitry associated with FOG. Secondly, Aβ may promote other proteins such as α-synuclein to misfold and accelerate disease progression; Thirdly, Aβ pathology is related to cognitive dysfunction, and cognitive dysfunction is a contributor for FOG. Those explanations were made based on brain Aβ plaque pathology. Although Aβ in CSF inversely correlated with Aβ plaque formation in brain reported by Fagan et al. [53], there is lack of direct evidence of brain Aβ pathology and FOG in this study.

### Medication use

Several studies recorded levodopa equivalent daily doses (LEDD) at baseline and explored whether it could be a factor to predict FOG development. In Forsaa EB’s study, higher LEDD was found to be an independent risk factor of FOG in a cohort of 234 PD patients with 12 years’ follow-up (OR = 1.30/100 mg, *P* = 0.009) [21]. Ou R et al’s study showed that the frequency of levodopa usage was higher in transitional freezers compared with non-freezers at baseline (72% vs.54%, *P* = 0.014) [23]. Two other studies found that transitional freezers had higher LEDD compared with non-freezers at baseline [18, 23], but failed to identify LEDD as a risk factor of FOG [18, 19, 23, 26]. However, clinicians practicing in the early years of levodopa introduction clearly felt they were witnessing a novel phenomenon that had not existed in the pre-levodopa era. Recently, Koehler PJ and his colleague reviewed films and medical textbooks before 1972. They chose 1972 as all patients in available films were levodopa naïve by then. They found that before the introduction of levodopa, FOG was not as common as it is nowadays and thus concluded that FOG episodes have increased after the introduction and long-term use of levodopa [54]. They gave explanations as follows: before levodopa was applied in clinic, motor symptoms were not well controlled and patients were too disabled to walk and demonstrate FOG; clinicians knew little about how to provoke FOG and seldom observe it; patients had short life span and disease duration might be too short to develop FOG; another possibility might be that FOG occurrence was induced by levodopa. According to the response to levodopa, FOG was classified as dopamine responsive FOG, dopamine resistant FOG and dopamine induced FOG (caused by administration of dopaminergic medication) [55]. It is hypothesized that peak-dose of oral levodopa caused dopaminergic over-stimulation, leading to dysfunction of forntal-subcortical circuits, which prompt “high-order” gait abnormalities such as FOG [56]. Future studies should evaluate whether and how chronic levodopa use increase the frequency of FOG events. Besides levodopa, Deprenyl treatment was strongly associated with a decreased risk for developing FOG [20]. Additionally, whether dopamine agonists (DA) increase the risk of developing FOG remains controversial [22, 32, 55, 57]. One study followed 248 early PD patients without FOG in which 128 patients developed FOG 3 years later. They found that the proportion of DA use was significantly higher in non-freezers than transitional freezers at baseline (60.8% vs.10.9%, *P* < 0.001). Binary logistic regression further established that not use DA was a risk factor of future FOG development [22]. However, several studies claimed that DA may increase the risk for developing FOG. A retrospective study showed that longer treatment with DA contributed to the appearance of FOG [32]. Bloem et al. also stated that based on their experience they had seen clear FOG induced by DA monotherapy [55]. In addition, ropinirole medication might deteriorate FOG in dopamine-responsive FOG patients with PD in a clinical trial [57].

## Limitation and recommendations for risk factor related researches for FOG

Firstly, FOG was assessed by subjective questionnaires in most studies. Showing the video to patients in testing new freezing of gait questionnaire (NFOGQ) [58] will help patients to understand FOG [55]. Using objective sensors to detect FOG will be more accurate to recognize FOG. Secondly, the follow-up was not long enough for some patients to develop FOG by the end of study, thus whether these risk factors could predict FOG over longer periods remains unclear. Thirdly, these studies didn’t differentiate “on” and “off” FOG. FOG is regarded to have different mechanisms under “on” and “off” medication states. Further studies are needed to explore the risk factors of specific type of FOG. Fourthly, these studies only explored the relationship between baseline parameters and FOG development except Ou R’s study [23], which calculated the changes of annual UPDRS. Thus future studies would better take disease progression rate into account by measuring the annual changes of scales, imaging or CSF markers. Meanwhile, some clinical domains should be assessed more comprehensively and objectively, such as using wearable sensors to evaluate gait disorders and balance impairments and neuropsychological battery to assess specific domain of cognitive dysfunction. Additionally, neuroimaging studies combining both structure and functional connectivity would better be explored prior to FOG onset and examined whether it can be used as an independent risk factor. At last, prospective studies including a larger sample of patients with longer follow-up period is strongly recommended.

### Drug treatment (The details of clinical trials are listed in Table 3)

#### Levodopa and levodopa-carbidopa intestinal gel (LCIG)

Robust evidence supports that dopamine replacement therapy with levodopa is the first choice for FOG treatment in PD [55]. Levodopa can significantly decrease the frequency and number of FOG episodes [3, 59]. FOG is more common and more prolonged during the off-state than during the on-state [3, 30, 96], and levodopa can reduce both off time and FOG severity [3]. To be note, the “threshold” to improve FOG is higher than the threshold to improve other motor signs in some patients, thus increased levodopa dosage without deterioration of other motor symptoms may potentially be effective [97]. However, there still exist numerous patients whose FOG is resistant to levodopa [30]. Continuous intra-jejunal infusion of LCIG is an effective advanced therapy for the treatment of motor fluctuations in PD [98, 99]. LCIG is administered via percutaneous endoscopic gastrostomy with a jejunal extension tube. The suspension form of carbidopa and levodopa can be delivered continuously into the intestine and plasma levodopa concentration can maintain stable. One retrospective study including 65 advanced PD showed that with LCIG treatment, FOG presented in 22% of patients at 1-year follow-up compared to 46% at the baseline [60]. Another retrospective study including 91 advanced PD with 18 ± 8.4 months’ follow-up also showed that gait disorders (freezing, festination, postural instability) improved in 61.4% of patients [61]. These results were supported by other clinical trials [62, 63]. Besides, long-term effectiveness of LCIG on FOG has also been demonstrated by two prospective studies. Sensi M et al. evaluated the long-term outcome in 28 PD patients, in which 17 patients reached the 24-month follow-up. Results showed that FOGQ at 6 months (*P* = 0.001) and 2 years (*P* = 0.03) were significantly lower than the pre-treatment condition [64]. Vijiaratnam N et al. also found that FOGQ improved 24% (11.9 ± 5.5 vs. 9.1 ± 5.3, *P* = 0.007) at 6 months in 25 PD patients [65]. Possible mechanism is that increasing “On” time by LCIG may lead to benefits on FOG when freezing episodes happen during the “Off” time. Importantly, two pilot studies found that LCIG can also improve levodopa-resistance FOG [56, 66]. One study including 5 PD with levodopa-resistance FOG who were treated with 24 h LCIG therapy for 6 months, and results showed median 360° turn time, fall frequency and FOGQ improved [66]. The other was a single section study including 7 PD with FOG. Evaluations were performed in “On” state either during oral levodopa (60–90 min after intake of usual morning levodopa dose) or LCIG (60–90 min after starting LCIG infusion). UPDRS item 14 freezing significantly improved with LCIG in all patients compared to oral levodopa (*P* = 0.026). FOGQ also significantly improved with LCIG (oral levodopa:19.1 ± 1.4, LCIG:10.4 ± 1.6, *P* = 0.017) [56]. There are several potential explanations for the beneficial effect of LCIG on levodopa-resistant FOG. First, LCIG is administered via the jejunum, which provides greater bioavailability of levodopa compared with oral administration. This may enable better absorption and more levodopa in the brain than administration of levodopa via the oral route. Second, continuous delivery of LCIG provides stable plasma levodopa concentrations and avoids the pulsatile dopamine stimulation in striatum by oral levodopa. Additionally, LCIG can also be delivered at night. Adding nocturnal LCIG infusion improves sleep and better sleep quality improves daytime motor functions including FOG [56, 66].

**Table 3:** Clinical trials of drug treatments for freezing of gait in Parkinson’s disease

Study reference	Participants	Study design	Treatment	Main findings	FOG subtype
**Levodopa**					
[3]	19 PD with FOG	Prospective, open-label, uncontrolled	Patients were examined during “Off” and “On” states that approximately 1 h after they took their regular morning dose of levodopa.	Levodopa significantly decreased frequency and the number of FOG episodes (Video recorded).	Unknown, but levodopa induced FOG was excluded
[59]	20 PD with FOG	Prospective, open-label, uncontrolled	Similar with the above study but took 1.5 times the usual levodopa dose	FOG improved (customized FOG score and FOGQ).	Unknown
**Levodopa-carbidopa intestinal gel (LCIG)**					
[60]	65 advanced PD	Observational, retrospective, a review of medical records	Mean duration of LCIG therapy was 3.7 years	FOG improved (FOG present only in 22% of patients at 1 year follow-up compared to 46% at baseline).	Unknown
[61]	91 advanced PD	Observational, retrospective, a review of medical records	Mean time of follow up of 18 ± 8.4 months	Gait disorders (freezing, festination, postural instability) improved in 61.4% of patients (three point scale).	Unknown
[62]	32 advanced PD with FOG	Observational, retrospective, a review of medical records	Mean duration of LCIG therapy was 2.59 ± 1.12 years	FOG that present in OFF condition and improved but did not disappear completely in ON condition can be further improved by LCIG (UPDRS freezing score).	31 patients with responsive FOG and one with resistant-FOG
[63]	177 advanced PD, in which 122 patients with FOG	Observational, retrospective, multi-center, cross-sectional, uncontrolled	Mean duration of LCIG therapy was 34.7 months, 80.8% of patients ≥12 months	FOG improved in 76.2% of patients (subjective assessment by clinicians).	Unknown
[64]	28 PD	Prospective, open label, uncontrolled	17/28 patients reached the 24-month follow-up	FOG improved (FOGQ)	Unknown
[65]	25 PD	Prospective, open label, uncontrolled	20 patients continued on treatment to 6 months.	FOG improved (FOGQ)	Unknown
[66]	5 PD with FOG	Prospective, open label, uncontrolled	24 h LCIG therapy, 6 months	360° turn time reduced, FOG improved (FOGQ) and fall frequency reduced	Resistant
[56]	7 PD with FOG	Prospective, open label controlled, unrandomized	Evaluations were performed in “On” state (60–90 min after taking the morning oral levodopa or LCIG).	FOG improved on LCIG (FOGQ and UPDRS freezing score)	Resistant
**Dopamine agonist**					
[67]	36 PD	Prospective, open label, uncontrolled	Pramipexole treatment for 3 months (started at 0.125 mg/day and increased to 1.5 mg/day) .	FOG improvement (FOGQ)	Unknown
[68]	111 PD, in which 54 patients with FOG	Prospective, open label controlled, unrandomized	Rotigotine transdermal patch (9-27 mg/day), pramipexole LA (1.5-4.5 mg/day), ropinirole CR (8-16 mg/day) for at least 6 months	FOG improvement in Rotigotine group (FOGQ)	48 patients with “Off” FOG and 6 with “Off and On” FOG
[69]	10 PD with FOG	Prospective, open label, uncontrolled	Acute test of subcutaneous apomorphine bolus in the morning at “off” state, without other medication	No improvement (subjective assessment)	FOG occur in both “Off” and “On” state
**Monoamine oxidase B inhibitors**					
**Selegiline**					
[70]	14 PD with FOG	Prospective, open label, uncontrolled	Addition or increase in dose of selegiline, average dose: 4.0 mg/day for 3 months	FOG improved in 7/14 patients (FOGQ)	Unknown
**Rasagiline**					
[71]	687 PD in which 278 patients with FOG	Prospective, double-blind, randomized, placebo-controlled	Oral rasagiline (1 mg once daily), entacapone (200 mg with every levodopa dose), or placebo for 18 weeks	FOG improved by Rasagiline (UPDRS-PIGD, UPDRS-freezing score)	Unknown
[72]	42 PD with FOG	Prospective, open label, uncontrolled, multicenter	1 mg rasagiline daily as an add-on therapy for 3 months	FOG improved after 1, 2 and 3 months of therapy (FOGQ)	Unknown
[73]	18 PD with FOG	Prospective, open label, uncontrolled,	1 mg rasagiline daily as an add-on therapy for 90 days	No overall improvement (Objective FOG counts and duration)	Resistant
**Methylphenidate (MPH)**					
[74]	69 advanced PD with FOG who had received STN- stimulation	Double-blind, randomized, Placebo-controlled	MPH (1 mg/kg per day) or placebo capsules for 90 days	MPH reduces FOG in both “off” and “on” levodopa conditions (FOGQ and the number of freezing episodes while taking walking trajectory)	Resistant
[75]	17 STN-stimulated patients with advanced PD and gait disorders	Prospective, open label, uncontrolled	A daily dose of 1 mg/kg of MPH three times daily) for 3 months, including a 1-month titration phase	3 months MPH improved FOG (number of FOG during Stand-Walk-Sit test)	Resistant
[76]	5 PD with FOG	Prospective, open label, uncontrolled	A single oral administration of 10 mg MPH. Reassessment 2 h later.	FOG improved (total walking time, total freezing time, number of freezing episodes and the non-freezing walking time during an “8” trajectory).	Responsive
[77]	17 PD with moderate gait impairment	Double-blind, randomized, placebo-controlled	MPH (maximum, up to 80 mg/day) or placebo for 12 weeks and crossed over after a 3-week washout.	No improvement (FOGQ)	Unknown
**Istradefylline**					
[78]	14 PD patients with FOGQ 12.14 ± 5.82	Prospective, open label, uncontrolled	20 mg Istradefylline daily for 1 month	FOG improved (FOGQ)	Unknown
[79]	31 PD patients with FOG	Prospective, open label, uncontrolled, multicenter	20 mg Istradefylline daily for 4 weeks, followed by 20 mg/day or an 40 mg/day for 8 weeks	FOG improved (FOGQ, NFOGQ, and MDS-UPDRS Part III (ON-state) gait-related items total score)	Unknown
**Antidepressants**					
[80]	52 PD with mild to severe depressive	Prospective, open label, randomized, controlled, multicenter	Paroxetine 20 mg/day or 25 mg/day; escitalopram 10 mg/day; duloxetine 40 mg/day; 8 weeks’ maintenance period and 2 weeks’ incremental period	FOG (FOGQ) and depression improved	Unknown
**L-DOPS, droxidopa**					
[81]	16 PD with FOG	Randomized, open label, controlled	L-DOPS and entacapone initially 100 mg per day, increase by 100 mg increments every 2 days up to 100 mg per each levodopa administration for 4 weeks	Co-administration of L-DOPS and entacapone improved FOG, yet entacapone or L-DOPS alone didn’t, and the improvement was found only in levodopa-resistant FOG (visual analogue scale, VAS)	14 patients with “On and Off FOG”; 2 patients with “Off” FOG
[82]	13 advanced PD with FOG	Prospective, open label, uncontrolled	L-DOPS initially 100 mg/day with a weekly increase of 100 mg up to 600-900 mg/day maintenance	FOG improved in more than half of patients (walk 10 m and return, subjective assessment)	Unknown
**Amantadine**					
[83]	11 PD with FOG	A retrospective chart review	Median 100 mg twice daily, and treatment duration was 20 months (range, 6-66 months).	Subjective self-reported improvement on FOG	Unknown
[84]	42 PD with FOG	Double-blind, randomized, placebo-controlled	200 mg/500 mL normal saline twice a day for 5 days.	No improvement (FOGQ)	50% patients with FOG at “On” state
[85]	15 patients with FOG including 6 PD	Prospective, open label, uncontrolled	200 mg in 500 cm3 of saline solution given over a 3-h period, twice a day for 2 days	Improvement in PD patients (FOGQ)	Resistant
[86]	10 PD with FOG	Randomized double-blind placebo-controlled, crossover	Placebo (normal saline) or amantadine (400 mg/day) were injected four times for 2 days, 52-h washout, then switched.	No improvement (FOGQ, UPDRS, 4 × 10 m walking test)	Resistant
**Atomoxetine**					
[87]	5 PD with FOG	Prospective, double-blind, randomized, placebo-controlled	10 mg daily and 10 mg increments up to 40 mg per day over 3 weeks.	No improvement (7 M Step test, FOGQ, Clinician’s Global Index of Change (CGIC), Gait and Balance Scale)	Resistant
[88]	10 PD with FOG	Prospective, open label, uncontrolled	40 mg daily for 2 weeks then increased to 40 mg twice daily for 4-week then reduced to 40 mg daily for 1 week	No improvement (FOGQ)	Resistant
**Acetylcholinesterase inhibitor**					
[89]	41 PD with dementia	Open label, randomized, controlled	Galantamine 4 mg twice daily for the first 4 weeks, and then 8 mg twice daily to the end of the 24 week trial period.	FOG improved (UPDRS freezing subitem)	Unknown
[90]	130 PD	Randomized, double-blind, placebo-controlled	Rivastigmine was uptitrated from 3 mg per day to the target dose of 12 mg per day over 12 weeks	FOG did not improve (episodes of FOG in the past month; NFOGQ)	Unknown
**Botulinum toxin**					
[91]	11 advanced PD with FOG	Randomized double blind placebo-controlled	BTX-A injection into each leg’s calf muscles, 150 IU per leg	No improvement (FOGQ, CGIC, UPDRS)	Off FOG
[92]	12 PD with FOG	Randomized double-blind placebo-controlled, crossover	BTX-A injection into calf muscles, 16.25 to 25 U /site, six injection sites per leg, 12-week washout, then switched	No improvement (FOGQ, diaries, TUG and “2-min walk test”)	Unknown
[93]	10 patients with FOG including 7 PD	Prospective, open label, uncontrolled	BTX-A injection into calf muscles, 3–6 sites per leg, 100-300 IU per session	FOG improved (CGIC)	3 patients with “Off” FOG; 2 with “On” FOG; 2 with “On and Off” FOG
[94]	20 PD, 10 PD with FOG and 10 PD without FOG	Prospective, open label, uncontrolled	BTX-A injection into tensor fasciae latae muscle, 50 U per leg	FOG improved (FOGQ)	Resistant
[95]	14 PD with FOG	Double-blind, placebo-controlled, randomized	BTX-B injection into calf muscles of the predominantly affected leg in freezing, 5000 U	No improvement (UPDRS, VAS, and Modified Webster Step-Seconds test)	Resistant

A total of 39 clinical trials were summarized. The number of participants and their FOG subtype, the type of study design, drug treatments strategies, assessment methods for FOG and main findings were provided in this table

*FOG* Freezing of gait, *PD* Parkinson’s disease, *LCIG* Levodopa-carbidopa intestinal gel, *FOGQ* Freezing of Gait-Questionnaire, *NFOGQ* New Freezing of Gait Questionnaire, *UPDRS* Unified Parkinson’s Disease Rating Scale, *PIGD* Postural instability/gait difficulty, *MPH* Methylphenidate, *L-DOPS* L-threo-3, 4-dihydroxyphenylserine, *VAS* Visual Analog Scale, *CGIC* Clinician’s Global Index of Change

#### Dopamine agonists (DA)

An open-label uncontrolled study evaluated transdermal patch rotigotine as monotherapy in untreated PD patients for 6 months and found that transdermal patch rotigotine improved all aspects of gait compared to baseline, including straight walking, gait initiation and turning [100]. Studies of DA as an adjunct to levodopa also found beneficial effects on gait speed in add-on therapy of apomorphine (sublingual) and pramipexole compared to levodopa alone [101, 102]. An open label study found 3 months pramipexole treatment as an add-on to levodopa or single administration, FOGQ was significantly improved from 5.4 ± 5.3 to 4.2 ± 4.1in 36 PD patients [67]. Another open label study compared effects of transdermal patch of rotigotine, pramipexole LA and ropinirole CR on FOG, and showed that transdermal patch of rotigotine treatment for 2 months significantly improve FOG [68]. In this study, 47 patients (24 patients with FOG, 51%) were treated with rotigotine (maintenance doses of 9-27 mg/day), 33 patients (16 patients with FOG, 48%) received pramipexole LA (1.5-4.5 mg/day) and 31 patients (14 patients with FOG, 45%) received ropinirole CR (8-16 mg/day). The FOGQ score was recorded during off time in patients with wearing off. FOGQ score were significantly decreased from 30.1 ± 1.8 at baseline to 21.0 ± 1.2 after 2 months rotigotine treatment, whereas pramipexole LA and ropinirole treatment did not alter FOGQ score compared to baseline. Such amelioration of FOG did not depend on reduction of off time after treatment, as off time improvements were similar among these treatments. One possibility is that rotigotine has higher binding affinity to D1/D5 receptors compared to pramipexole and ropinirole. The distinct pharmacological profile and 24-h steady hemodynamics of rotigotine might play a role in the therapeutic mechanism of FOG in PD patients with wearing off. Although subcutaneous continuous apomorphine infusion providing continuous dopaminergic stimulation similar to LCIG, the results of a clinical trial were disappointing [69]. Both the rotigotine transdermal patch and continuous apomorphine infusion are more convenient and reversible than LCIG; therefore, future research is needed to clarify whether they have beneficial effects on refractory FOG.

#### Monoamine oxidase B inhibitors (MAO-B inhibitor)

Clinical trials have shown that selegiline was associated with reduced risk of future FOG [20, 103]. Lijima et al. used wearable sensors to record the daily walking profiles of 14 PD patients before and after selegiline add-on therapy, and used FOGQ to evaluate their FOG. Results revealed that FOGQ and UPDRSIII significantly improved after 3 months selegiline add-on or selegiline dose-increasing therapy (FOGQ, 9.2 ± 4.9 vs 7.9 ± 5.1, *P* = 0.01; UPDRSIII 18.1 ± 9.0 vs. 13.1 ± 10.0, *P* < 0.005). Hypokinesia index was also achieved by a small device that attached to the waist of the patients that measured three dimensionally the accelerations accompanied by limb and trunk movements and accelerations during gait. The results showed that improvements in hypokinesia and gait disorders did not occur in parallel. They suggested the beneficial effect was due to selegiline increased the synthesis of phenylamine, which might facilitate the release of noradrenaline. Hypokinesia of limb and trunk movements were not improved in this study indicating the beneficial effect exerted by selegiline add-on therapy was not by dopaminergic pathway [70]. Rasagiline, as an effective monotherapy in early PD and add-on therapy in PD patients with motor fluctuations [71, 104–106], has also been studied on FOG. In the LARGO study [71], a prospective, double-blind, randomized, placebo-controlled study enrolled 687 PD patients, in which 278 patients had FOG. Oral rasagiline (1 mg once daily) provided a significant improvement in UPDRS-PIGD (*P* = 0.034) and UPDRS-freezing scores (*P* = 0.045). In addition, in an open-label, multicenter study, 42 patients with FOG were treated with 1 mg rasagiline daily as an add-on therapy, and FOGQ was assessed as primary outcome [72]. Results showed that patients treated with rasagiline had a statistically significant decrease in FOGQ score after 1, 2 and 3 months of therapy. However, the proportion of levodopa-unresponsive FOG in these studies was unclear. Only one study investigated the effect of MAO-B inhibitor on levodopa unresponsive FOG. This study found that 6 of 14 PD patients with intractable FOG showed reduction in FOG number and duration after a 90-day course of 1 mg daily rasagiline add-on therapy [73].

#### Methylphenidate (MPH)

MPH influences both the dopaminergic and norepinephrine systems and thus is supposed to play a positive role in FOG treatment. One multicenter, parallel, double-blind, placebo-controlled, randomized study enrolled 69 PD patients who had severe gait disorders and FOG despite receiving an optimized, stable dose of levodopa and subthalamic nucleus (STN) stimulation. The results showed that MPH (1 mg/kg per day) treatment for 90 days was effective on levodopa-resistant FOG. In this study, FOG was assessed by FOGQ and the number of freezing episodes while taking walking trajectory [74]. Another study including17 advanced PD patients who presented gait disorders, despite their use of optimal dopaminergic doses and STN stimulation also showed that a daily dose of 1 mg/kg of MPH for 3 months significantly improved gait in the absence of levodopa as assessed by the walking speed, the number of steps and the number of freezing episodes [75]. Besides, one study tested the immediate effect of low dose MPH on five advanced PD and found the beneficial effect of MPH on FOG. The patients were tested during “off” state before and 2 h after the intake of 10 mg MPH while walking an “8 trajectory”. The total walking time, total freezing time, number of freezing episodes and the non-freezing walking time were assessed and all these parameters were improved [76]. But the result of another 6-month placebo-controlled, double-blind, crossover study was disappointing [77]. Twenty-seven subjects with PD and moderate gait impairment were randomly assigned to MPH (maximum, up to 80 mg/day) or placebo for 12 weeks and crossed over after a 3-week washout. Results showed MPH did not improve FOG as assessed by FOGQ and the freezing diary. Further well-designed studies were needed to explore the effectiveness of MPH on FOG.

#### Istradefylline

Caffeine, which is an adenosine receptor antagonist, has been shown to be effective for the treatment of FOG [107]. Istradefylline is a selective adenosine A2A receptor antagonist, which has also been found to be useful in reducing “off” time in PD [108], thus be tested for FOG therapy. Fourteen PD patients were treated with 20 mg of Istradefylline in the morning. FOGQ scores were significantly decreased 1 month after Istradefylline treatment (9.79 ± 7.16) when compared to those before the intervention (12.14 ± 5.82, *P* = 0.030) [78]. Recently, Mutsumi Iijima et al. conducted a multicenter, open-label, prospective interventional study which evaluated changes in total gait-related scores of the MDS-UPDRS II/III, FOGQ and NFOGQ in 31 PD patients treated with istradefylline. Patients with advanced PD who were treated with levodopa preparations and had wearing-off symptoms and gait disorders complicated with FOG were enrolled. Istradefylline was orally administrated for 12 weeks at 20 mg/day for the first 4 weeks, followed by 20 mg/day or an increased dose of 40 mg/day for 8 weeks. Istradefylline significantly improved MDS-UPDRS Part III (ON-state) gait-related items total score after 12 weeks treatment compared with baseline (− 1.1 ± 2.0, *P* = 0.007), FOGQ (− 2.0 ± 3.1, *P* = 0.002), and NFOGQ (− 2.2 ± 5.1, *P* = 0.026) [79]. Istradefylline showed promising in treating FOG, but further studies involving larger patient numbers and randomized, controlled design are needed.

#### Antidepressants

Depression is found to be a risk factor for FOG development and limbic system has been proven to be involved in FOG pathophysiology, which were discussed previously. However, only one study has studied the effect of antidepressants on FOG therapy. This prospective, multicenter, open-label, randomized study assigned PD patients with mild to severe depression into selective serotonin reuptake inhibitors (SSRIs) (paroxetine or escitalopram, 27 enrolled, 25 analyzed) or serotonin and norepinephrine reuptake inhibitors (SNRIs) (duloxetine, 28 enrolled, 27 analyzed) group. The mean changes (from baseline) of FOGQ scores at 6 and 10 weeks were statistically significant in both the SSRI (6 weeks, − 2.2, *P* = 0.018; 10 weeks, − 2.9, *P* = 0.012) and SNRI (6 weeks, − 2.1, *P* = 0.047; 10 weeks, − 3.4, *P* = 0.010) groups [80], indicating that FOG was significantly improved. No significant differences were observed between the SSRI and SNRI groups. Depression symptom was also improved. Further studies are needed to evaluate the effects of antidepressants on FOG.

#### L-threo-3, 4-dihydroxyphenylserine ((L-DOPS, droxidopa)

One open label, controlled study randomized 16 PD patients with FOG into L-DOPS co-administered with entacapone group (*n* = 6), entacapone alone group (*n* = 5), and L-DOPS alone group (*n* = 5) in addition to their original medication. Results showed that the combination of L-DOPS and entacapone as an add-on therapy significantly improved FOG based on the visual analogue scale at 4 weeks compared with baseline while L-DOPS alone did not. The improvement was observed only in levodopa-resistant FOG patients, suggesting that dysfunction of noradrenergic neurons plays a significant role in levodopa-resistant FOG [81]. A prospective, open label, noncontrolled study including 13 PD with FOG, found that L-DOPS initially 100 mg/day with a weekly increase of 100 mg up to 600-900 mg/day maintenance, FOG improved in half of patients [82].

#### Amantadine

A retrospective study reviewed PD patients who received amantadine specifically for FOG and found that 10 of 11 patients reported subjective improvement in FOG after initiating amantadine. Median amantadine dosage was 100 mg twice daily, and treatment duration was 20 months (range 6-66 months). Four patients reported reduction in benefit after 4 months, indicating that this effect may be transient [83]. Although the mechanism of amantadine in PD is unclear, dopaminergic and non-dopaminergic mechanisms are both involved. Additionally, the effect of intravenous amantadine on FOG has been explored, and the results were inconsistent [84–86]. In Kim et al’s open label study, 6 PD patients with intractable freezing was administrated with intravenous amantadine (200 mg in 500 cm^3^of saline solution given over a 3-h period), twice a day for 2 days along with the pre-existing anti-parkinsonism medication. Results showed a marked improvement on FOG (FOGQ score ≤ − 4) in 5 of 6 PD patients [85]. However, two double-blind, randomized, placebo-controlled studies including 42 PD and 10 PD patients with FOG respectively found that intravenous amantadine had no beneficial effect on FOG [84, 86].

#### Atomoxetine

Atomoxetine is a selective norepinephrine reuptake inhibitor that enhances noradrenergic transmission, thus it is supposed to be effective for FOG. However, a randomized double-blind, placebo-controlled study enrolled 5 patients did not find its efficiency on FOG [87]. Another eight-week open label study enrolled 10 PD patients with dopamine-unresponsive FOG also failed to demonstrate its efficiency [88].

#### Acetylcholinesterase inhibitor

An underlying loss of cholinergic function contributes to freezing [109], thus the effect of acetylcholinesterase inhibitors on FOG has been tested. One open controlled study included 41 patients with PD with dementia and randomized patients to a galantamine treatment group in which patients received galantamine in addition to ongoing treatment (21 patients) and a control group (20 patients) who continued ongoing treatment. Results showed 24-week galantamine (8 mg twice daily) add-on therapy significantly improved freezing assessed by UPDRS freezing subitem (3.0 ± 0.5 to 2.3 ± 0.4, *P* = 0.03) [89]. But recently the double blind placebo-controlled study did not find FOG improvement after acetylcholinesterase inhibitor rivastigmine treatment [90].

#### Botulinum toxin (BTX)

Two double blind placebo-controlled studies using subjective and objective measures found no significant FOG improvement with BTX-A injections into calf muscles [91, 92], albeit the beneficial effect has been observed previously in two open label studies [93, 94]. Apart from BTX-A, the effect of BTX-B on FOG was also disappointing [95], thus this approach is therefore discouraged.

## Summary of pharmacological treatments for FOG

In this section, a total of 39 clinical trials were included in this review, but high quality RCT studies were rare. Meanwhile, there was no meta-analysis in this field. Despite the limited effectiveness of current medications for FOG, there were still some clinical studies that showed promise for some drugs. Levodopa is the first choice for FOG treatment in PD [3, 55, 59]. Besides, a total of eight studies containing both retrospective and prospective open-label studies all found that LCIG improved FOG significantly [56, 60–66]. LCIG is an invasive approach, which limits its clinical application to some extent. One prospective, open label, controlled study showed transdermal patch of rotigotine treatment significantly improved FOG [68]. This promising result needs to be examined by further studies. One open-label study supported selegiline and one double-blind, RCT study and one open-label study supported rasagiline as add-on treatments for FOG [70–72]. One multicenter, parallel, double-blind, placebo-controlled, randomized study found that MPH improved levodopa-resistant FOG in those patients who has already received an optimized, stable dose of levodopa and STN stimulation [74, 75]. Therefore, for PD patients who had received STN-Deep brain stimulation (DBS) but still with FOG, 1 mg/kg/day MPH could be tried. Recently, two pilot open-label studies showed Istradefylline was effective on FOG treatment [78, 79], and one prospective, open label, randomized, controlled, multicenter study found SSRIs (paroxetine or escitalopram) or SNRIs (duloxetine) improved FOG significantly [80].

Continuous subcutaneous apomorphine infusion is reversible and more convenient than LCIG, but the result of one small study was disappointing. The efficiency of subcutaneous apomorphine infusion on FOG needs to be tested in future studies. It is worth noting that several studies supported that the DA increased the risk for FOG [32, 55, 57], physicians should take caution while prescribing DA for PD patients with FOG. There was lack of strong evidence that oral amantadine can improve FOG and two double-blind, randomized, placebo-controlled studies found that intravenous amantadine had no beneficial effect on FOG [84, 86]. L-DOPS plus entacapone were shown to be effective in improving FOG, but whether L-DOPS alone could improve FOG needed further studies [81, 82]. An underlying loss of cholinergic function contributes to freezing, but the recently double blind placebo-controlled study did not find FOG improvement after acetylcholinesterase inhibitor rivastigmine treatment [90], although a previous open label controlled study showed positive results under another acetylcholinesterase inhibitor galantamine treatment. Botulinum toxin and Atomoxetine are ineffective according to the clinical evidence.

Despite some treatments showed promising results, it is still difficult to treat FOG in PD, especially those levodopa resistant FOG. We cannot conclude one drug was effective based on limited clinical trials without meta-analysis or high quality RCTs. Similarly, we cannot deny one because of limited studies that have not shown the effectiveness. Future multicenter, large sample, RCT studies are needed to test the effects of these drugs, especially the promising ones.

## Non-pharmacological therapy

### Spinal cord stimulation (SCS)

Recently, one open-label, nonrandomized pilot study investigated the safety and efficacy of SCS on gait in 4 patients with advanced PD previously treated with STN-DBS. Paddle electrodes with 3 columns of contacts were implanted in the epidural space of the upper thoracic spine (T2-T4). Results showed 300 Hz/90us SCS induced a significant improvement in gait and FOG. FOGQ improved from 17.8 ± 0.9 at baseline to 7.8 ± 0.9 at 6-months(*P* < 0.001) [110]. The beneficial effect of SCS was also found in another open-label, nonrandomized pilot study, 5 PD participants with significant gait disturbances and FOG despite optimization of dopaminergic medication underwent mid-thoracic SCS (T8-T10, 300-400 us/30-130 Hz). Four of the five participants reported FOG occurred less frequently [111]. The mechanisms involved in SCS modulating gaits are unclear. It has been reported that SCS may influence neuronal firing in SMA, a key hub for controlling gait initiation [112, 113]. Another theory said that SCS might disrupt the aberrant inhibition of the globus pallidus internus onto thalamus and SMA [114, 115]. Anticipatory postural adjustment (APA) is required in advance of each step forward for normal gait. During freezing episodes, the intention to walk is uncoupled from the triggering of APA, and then consequent failure of the forward movement happened. SCS improved APA [116] and APA is found to be modulated by SMA [112], thus it has been supposed that SCS improved FOG probably by modulating SMA to improve APA and facilitate gait initiation [117].

### Deep brain stimulation (DBS)

#### STN-DBS high frequency stimulation (HFS)

A meta-analysis summarized the short and long-term effects of bilateral STN-DBS with HFS (usually 130 Hz) on gait and FOG in PD [118]. Seven studies with available data of FOG (UPDRS item 2.14) were included [119–125]. The results showed that gait and FOG could be improved for more than 4 years in the Med-Off/Stim-On condition. However, no beneficial effect was found for the Med-On/Stim-On compared with Med-ON/Stim-Off. This is probably due to the selection criteria for DBS candidates was levodopa responsiveness of global motor performance as well as gaits. These results confirmed the lack of a synergistic effect of medication and DBS [118]. Other studies also reported that STN-HFS improved FOG in most patients [126–128]. However, STN-DBS worsened or induced the new appearance of FOG and/or gait akinesia during the immediate postoperative period in some patients [129, 130]. Such paradoxical deterioration of gait and akinesia is rare, and this side effect is probably related to misplaced contacts [131]. Therefore, when levodopa-responsive FOG patients is complicated by dose related side effects of levodopa treatment, high frequency STN-DBS can be considered. Meanwhile, antidepressant treatment can be tried if depression exists, as depression may negatively affect the outcome of HFS on FOG [132].

#### STN-DBS low frequency stimulation (LFS)

LFS (most commonly 60 Hz) STN-DBS has been tried to treat PD patients with FOG and have shown its short-term or even long-term beneficial effects on improving FOG and other axial symptoms compared with HFS [133–135], although some studies argued that there was no significant difference between HFS and LFS for controlling FOG [136, 137]. Currently, there are lack of meta-analysis to examine efficiency of low frequency STN-DBS on FOG, thus caution should be used in interpreting the results of these clinical studies and drawing conclusions. However, LFS should be recommended in STN-DBS patients with refractory axial symptoms (particularly FOG) at HFS, as long as tremor doesn’t worsen significantly with LFS [138–140].

#### PPN-DBS

One randomized double-blinded study evaluated the long-term effect of PPN–DBS with a follow-up period for 24–48 months and reported that the benefits on FOG and falls lasted at least 4 years in four of the six patients [141]. On behalf of the Movement Disorders Society PPN–DBS Working Group, Thevathasan et al. reviewed the literature from 1990 to 2017 comprising fewer than 100 cases and summarized the outcomes of PPN-DBS application in patients with PD [142]. Although firm conclusions cannot be drawn on therapeutic efficacy, the literature suggested that PPN-DBS could improve medication refractory gait freezing and falls [143–148]. These cases were from different surgical centers; therefore, there was great variability in clinical methodology. Caudal or rostral PPN, unilateral or bilateral implantation, high or low frequency stimulation are factors that may affect the outcome. Thus, consensus about the optimal methodology is needed.

#### Dual-site DBS

A crossover, double-blind, randomized controlled trial enrolled 12 PD patients with gait and balance impairment resistant to optimized dopaminergic and STN-DBS treatment found that combined stimulation of the STN and substantia nigra pars reticulata (SNr) using interleaved stimulation of both sites at a high frequency of 125 Hz improved FOG at 3-month follow-up [149]. In another randomized, crossover pilot study, Valldeoriola and colleagues modified this approach by co-stimulating STN and SNr at different frequencies in 6 patients, and investigated the effects on FOG and other PD motor symptoms [150]. The results suggested that combined HFS-STN and LFS-SNr stimulation had the best effect on various measures of FOG, compared to stimulation of a single target alone. However, no comparative data between low- and high-frequency SNr stimulation (neither SNr alone nor combined with STN) is available, whether a low frequency at SNr would be superior over high frequency remains unclear.

### Noninvasive vagus nerve stimulation (VNS)

Recently, it has been reported that VNS administered for 10 days improved locomotion in a rodent model of PD [151]. Thus, an observational, open-label, pilot study explored the effect of single-dose, noninvasive VNS on gait pattern and FOG in 12 patients with FOG. A total of 2 VNS treatments were applied to the left vagus nerve in left side of the neck below the mandibular angle, medial to the sternocleidomastoid muscle and lateral to the larynx, with an interval of 15 min between 2 VNS treatments. Each VNS treatment consisted of 120 s of stimulation. Assessments were performed just before and 15 min after the application of VNS. Video analysis showed that VNS significantly improved the number of steps taken while turning [152]. The mechanism was still unknown. One possibility is that locus coeruleus noradrenergic neurons degenerate prior to substantia nigra dopaminergic neurons in PD, and VNS may activate locus coeruleus neurons [151]. A multicenter, double-blind, placebo/sham-controlled randomized trial of noninvasive VNS in patients with PD are needed.

### Transcranial magnetic stimulation (TMS)

TMS induces electrical current generated through a rapidly changed magnetic field and activates cortical neurons located up to 2–3 cm beneath the scalp. Recently, one meta-analysis enrolled 7 RCTs including 102 participants, where 6 studies using repetitive TMS (rTMS) and one using dual-mode and dual-site (rTMS + tanscranial direct current stimulation (tDCS)). Results showed that rTMS had a beneficial effect on FOG in PD patients assessed by FOGQ and turning time. However, subgroup analysis according to stimulation site showed neither motor cortex stimulation nor frontal cortex stimulation had beneficial effect on FOG [153]. Other stimulation site has also been explored. One recent RCT including 30 PD patients with FOG showed that 10 sessions of high-frequency (10 Hz) rTMS over the SMA had beneficial effects on FOG in PD patients [154]. This study also found that the beneficial effects could last at least 4 weeks after stimulation. This result was consistent with Kim’s results, where Kim et al. [155] reported significant improvements after 2 sessions of high-frequency SMA stimulation in 12 PD patients, but not after motor cortex stimulation. These results suggested that SMA stimulation may be a more-appropriate target in PD patients with FOG, which need to be confirmed by future studies.

### Transcranial direct current stimulation (tDCS)

In general, anodal tDCS facilitates cortical excitability. In a crossover, double-blind, randomized, sham-controlled study including 10 PD patients with levodopa-resistant FOG, five sessions of 2 mA anodal tDCS on primary motor cortex showed benefits on FOG and motor performance after tDCS [156]. However, another crossover double-blind, randomized, sham-controlled study applied one session multibipolar tDCS electrodes stimulating only primary motor cortex in PD patients with FOG didn’t improve FOG. But after stimulating both primary motor cortex and left dorsolateral prefrontal cortex, the performance in gait-provoking test, stroop, and time up and go tests were improved [157]. Besides, another double-blinded crossover randomized, sham-controlled study including 10 PD with FOG showed a single dose of anodal tDCS over the SMAs did not improve self-initiated gait in PD and FOG [158].

### Physiotherapy

Cues are the most well-known and widely studied behavioral approaches for ameliorating FOG. Cue is defined as using external temporal or spatial stimuli to facilitate movement (gait) initiation and continuation [159]. Cues are classified into visual, auditory or somatosensory cue based on delivery modality classification. The remarkable responsiveness of PD patients to external cues has inspired several recent studies to evaluate the potential of technology-based wearable systems for improving FOG. For example, laser-shoe, a new form of continuous ambulatory cue has been developed recently. The laser-shoe is equipped with a line-generating laser activated through the loading of the body weight onto the switch upon heel contact during the gait cycle. The laser line appears orthogonally in front of the patient’s contralateral foot. Thus the cues are tuned exactly to the step frequency of the patient. A total of 21 patients with PD and FOG were tested in a controlled gait laboratory, both “off” and “on” medication. Cueing using laser shoes was associated with a significant reduction in the number and duration of FOG episodes, both “off” and “on” medication [160]. Besides, in McCandless et al’s study, rhythmic tactile stimulation (pulsed vibration) was provided at an adjustable tempo of 10 to 280 beats/minute via a vibration clip attached anteriorly over the right side of the user’s pelvis [161]. Results showed an immediate positive effect on the mean percentage of Off-FOG episodes during the walking task in 20 PD patients. In Zhao et al’ s study [162], they used Google glass to provide three audiovisual cues: bone-conduction allowed users to hear the rhythmic auditory sound through vibrations of Google Glass; flashing light (LED) caused the screen to rhythmically flash on and off; the optic flow generated vertically oriented lines on both sides of the screen that moved forward at a fixed speed. Zhao et al. carried out measurements on 12 participants and observed a significant immediate effect of auditory cueing on the mean frequency of FOG episodes during walking tasks with 360° turn. Nine of 12 participants were willing to use Google Glass at home to address FOG. Recently, Sweeney et al. reviewed wearable cueing devices delivering visual, auditory or vibration cue and addressing FOG in PD in recent years, and found that cueing was generally effective and promising [163]. However, it should be noted that some devices although seems promising, efficacy of various cueing devices need to be further tested outside the home or laboratory and in a larger PD patients with FOG.

Apart from cues, treadmill training [164–168], obstacle aquatic training [169], supervised slackline training [170], walk-bicycle [171], and action observation [172, 173] have also shown positive effects on FOG. Recently, Cosentino C et al. [174] performed a systematic review and meta-analysis to evaluate the effectiveness of physiotherapy intervention on FOG symptoms. A total of 19 studies, including 913 patients, were enrolled and FOGQ was used as the primary outcome measure. This review provides evidence for short-term effectiveness of physiotherapy in improving FOG compared with no treatment (effect size = − 0.28 [− 0.45, − 0.11], *P* = 0.001) or usual care (effect size = 0.43 [− 0.65, − 0.21], *P* < 0.0001). These results seem to be maintained at the follow-up examinations (effect size = − 0.52 [− 0.78, − 0.26]; *P* = 0.001). Furthermore, among these various interventions, action observation, treadmill combined with cueing, and prolonged homebased exercise trainings are able to impact on FOG more than other approaches.

## Summary of non-pharmacological treatments for FOG

In this section, invasive stimulation including DBS and SCS, noninvasive brain stimulations including tDCS, rTMS, noninvasive VNS, and physiotherapy including cues and other training strategies have been reviewed and summarized. A meta-analysis containing 7 studies revealed that HFS STN-DBS improved Med-off gait and FOG, and this beneficial effect could maintain for more than 4 years [118]. Although there was lack of strong evident that LFS STN-DBS could improve FOG, some literature suggested that LFS should be recommended in STN-DBS patients with refractory axial symptoms (particularly FOG) as long as tremor did not worsen significantly with LFS [138–140]. PPN was a new promising target for DBS to address FOG in recent years. Thevathasan et al. reviewed the literature from 1990 to 2017 and suggested that PPN-DBS could alleviate medication refractory gait freezing and falls [142]. In addition, small sample pilot studies showed SCS [110, 111] and dual site DBS such as combined stimulation of the STN and SNr [149, 150] significantly improved FOG. These promising results need to be examined by further RCT studies in a larger sample. Of noninvasive brain stimulation, tDCS and rTMS have been examined for FOG treatment in PD. Pilot studies showed that SMA may be a more-appropriate target for rTMS in PD patients with FOG [154, 155]. Besides, one recent study showed that VNS significantly improved FOG in 12 PD patients [152]. Multicenter, double-blind, placebo/sham-controlled randomized trials are needed to confirm theses exciting and promising findings. Of physiotherapy, wearable cueing devices was generally effective and promising [163]. However, the efficacy of various cueing devices need to be further tested outside the home or laboratory and in a larger PD patients with FOG. Apart from cues, a systematic review and meta-analysis found that action observation, treadmill combined with cueing, and prolonged homebased exercise trainings were able to impact on FOG more than other approaches [174].

## Conclusions

FOG is a common, disabling symptom of PD, affecting patients’ independence and quality of life, and often contributing to wheelchair use and falls. Risk factors of FOG are crucial for screening FOG and are helpful to understand mechanism of FOG as well. Demographic information, motor symptoms, non-motor symptoms, neuroimaging, fluid parameters, and medication use were explored. Among them, gait disorders, PIGD phenotype, lower striatal DAT uptake were found to be independent risk factors of FOG with consistent evidence. Other risk factors with contradictory or limited evidence need to be tested in future studies. How to optimize the combination of these risk factors and further improve the accuracy of FOG prediction is an important problem to be solved. Several pharmacological treatments are available and effective in terms of reducing the number and duration of freezing episodes. Dopamine replacement therapy with levodopa is the first choice for FOG treatment in PD. LCIG showed beneficial effect on FOG with consistent findings, but it is an invasive approach, which limits its clinical application to some extent. Moreover, Istradefylline and rasagiline showed promising in treating FOG, but further studies involving larger patient numbers and randomized, controlled design are needed, especially for those levodopa resistant FOG. Recently, scientists have introduced some promising non-pharmacological treatments. Several novel therapeutic strategies seem to be effective, such as rTMS over SMA, dual-site DBS, SCS and VNS. Of physiotherapy, wearable cueing devices seem to be generally effective and promising, but efficacy of various cueing devices need to be further tested outside the home or laboratory and in a larger PD patients with FOG.

## Data Availability

All the data mentioned in this article are available on published article.

## Abbreviations

FOG: Freezing of gait
PD: Parkinson’s disease
PPMI: Parkinson’s Progression Markers Initiative
FOGQ: Freezing of Gait-Questionnaire
NFOGQ: New freezing of gait questionnaire
MDS-UPDRS: Movement Disorder Society-Sponsored Revision of the Unified Parkinson’s Disease Rating Scale
PIGD: Postural instability and gait difficulty
PMSF: Pontomedullary reticular formation
MLR: Mesencephalic locomotor region
PPN: Pedunculopontine nucleus
SMA: Supplementary motor area
PM: Premotor area
RBD: REM Sleep Behavior Disorder
RBDSQ: RBD screening questionnaire
LEDD: Levodopa equivalent daily doses
DAT: Dopamine transporter
CSF: Cerebrospinal fluid
WMH: White matter hyperintensities
TD: Tremor-dominant
HR: Hazard ratio
OR: Odds ratio
ESS: Epworth Sleepiness Scale
LCIG: Levodopa-carbidopa intestinal gel
DA: Dopamine agonists
MAO-B inhibitor: Monoamine oxidase B inhibitors
MPH: Methylphenidate
L-DOPS: L-threo-3, 4-dihydroxyphenylserine
SSRIs: Selective serotonin reuptake inhibitors
SNRIs: Serotonin and norepinephrine reuptake inhibitors
DATATOP: Deprenyl and tocopherol antioxidative therapy of parkinsonism
BTX: Botulinum toxin
STN: Subthalamic nucleus
SCS: Spinal cord stimulation
APA: Anticipatory postural adjustment
HFS: High frequency stimulation
LFS: Low frequency stimulation
DBS: Deep brain stimulation
VNS: Vagus nerve stimulation
TMS: Transcranial magnetic stimulation
tDCS: Transcranial direct current stimulation
SNr: Substantia nigra pars reticulata
